# Keratitis due to *Aspergillus flavus*: Clinical profile, molecular identification of fungal strains and detection of aflatoxin production

**Published:** 2010-05-11

**Authors:** George Leema, Jayaraman Kaliamurthy, Pitchairaj Geraldine, Philip A. Thomas

**Affiliations:** 1 Department of Animal Science, School of Life Sciences, Bharathidasan University, Tamil Nadu, India; 2 Institute of Ophthalmology, Joseph Eye Hospital, Tamil Nadu, India

## Abstract

**Purpose:**

To document the clinical profile of patients with keratitis due to *Aspergillus flavus* and to elaborate on differences in the aflatoxin-producing potential of keratitis strains versus environmental strains of *A. flavus*.

**Methods:**

Over a 6-month period, strains of *Aspergillus flavus* were isolated in culture from corneal scrape or biopsy material of patients who presented with suppurative keratitis (clinical isolates). The strains were confirmed to be *A. flavus* by molecular methods (amplification of the internal transcribed spacer 2 [ITS 2] region and direct sequencing followed by comparative GenBank analysis). The aflatoxin-producing potential of each strain was determined by thin-layer chromatography. The ability of each strain to form sclerotia in Czapek-Dox agar (CDA) after 7 days incubation at 30 °C in the dark and to produce a beige ring in yeast extract sucrose agar supplemented with methyl β-cyclodextrin and sodium desoxycholate (YESD medium) after 3 days incubation at 30 °C was also assessed. For comparison, the tests were also run on 10 strains of *A. flavus* (identity confirmed by molecular methods) collected from local farming areas (environmental isolates).

**Results:**

Aflatoxin B1 was detected in 16 (80%) of 20 culture filtrate or mycelial homogenate samples of the clinical isolates (mean concentration: 366.7±125.4 parts per billion [ppb]) but in only eight (40%) of 20 samples of environmental isolates (mean concentration: 306.6±125.4 ppb). Seven of the eight aflatoxin-producing clinical isolates and two of the four aflatoxin-producing environmental isolates formed sclerotia (>400 μm) and a beige ring in culture.

**Conclusions:**

Aflatoxin B1 was detected in a significantly higher percentage of growth samples of clinical isolates (80%) than growth samples of environmental isolates (40%) (χ^2^=6.667; p=0.0098); the therapeutic implications of this finding require further study. The production of sclerotia and a beige ring in culture appear to be useful markers of aflatoxin-producing potential in strains of *A. flavus* isolated from keratitis.

## Introduction

*Aspergillus flavus* is the name now used to describe a species as well as a group of closely related species of aspergilli [1]. *A. flavus* is second only to *Aspergillus fumigatus* as a cause of human invasive and non-invasive aspergillosis [2-4]. *A. flavus* is also an important cause of keratitis [5] and is reported in some studies to be the most frequent *Aspergillus* species causing keratitis [6-8].

*A. flavus* is able to produce potent mycotoxins, known as aflatoxins that are potentially harmful to humans and animals. Of the aflatoxins, aflatoxin B1 is particularly important, since it is the most toxic and potent hepatocarcinogenic natural compound ever characterized [9]. The ability of strains of *A. flavus* to produce aflatoxins is reported to be highly variable; several strains are non-toxigenic because aflatoxin synthesis may become unstable in these fungi [10]. Moreover, the production of aflatoxins is influenced by various environmental conditions such as temperature [11-13], pH [14-16], and nutrient sources [17-19].

Aflatoxins are known to be acutely and chronically toxic to animals, including humans, and can cause acute damage to, or cirrhosis of, the liver, induce tumors and produce teratogenic effects [20,21]. Interestingly aflatoxin B1 and/or its metabolites appear to localize in the melanin pigment of the eye, according to observations made in the eyes of mice [22,23], cows [23], rainbow trout fish [24] and sheep [25] following the administration of radiolabelled aflatoxin B1 by various routes; localization of radiolabelled aflatoxin B1 in the vitreous [24] and the palpebral and bulbar conjunctiva [25,26] have also been noted. When aflatoxin B1 was injected into chick embryos, anophthalmia was one of the malformations noted [27]. Specific toxic effects of aflatoxins in the cornea were demonstrated in chicks that had been administered aflatoxin orally; haziness of the cornea and separation of corneal lamellae, in addition to infiltration by polymorphonuclear leucocytes, were observed [28].

Although *A. flavus* is an important cause of keratitis, and aflatoxins are known to be produced by *A. flavus,* there is a paucity of data regarding possible aflatoxin production by *A. flavus* strains isolated from patients with keratitis. However, the results of one unpublished study (M. Saraswathy, 2006, PhD thesis, Bharathidasan University, Tiruchirapalli, India) suggested that aflatoxin production occurred more frequently in isolates of *A. flavus* from patients with keratitis than it did in isolates of *A. flavus* from the environment. These findings are interesting since filamentous fungi causing keratitis, such as species of *Aspergillus* and *Fusarium*, are known to ultimately originate from the environment.

In the present report, the clinical profile of a series of patients with culture-proven *A. flavus* keratitis is briefly documented. Molecular methods were used to confirm the identity of the fungal strains isolated as *A. flavus.* More importantly, the potential of the keratitis *A. flavus* strains to form aflatoxin, and associated markers of aflatoxin production, in culture has been compared with similar potential of *A. flavus* strains isolated from the environment.

## Methods

### Patients and clinical strains of fungi

**Figure 1:**
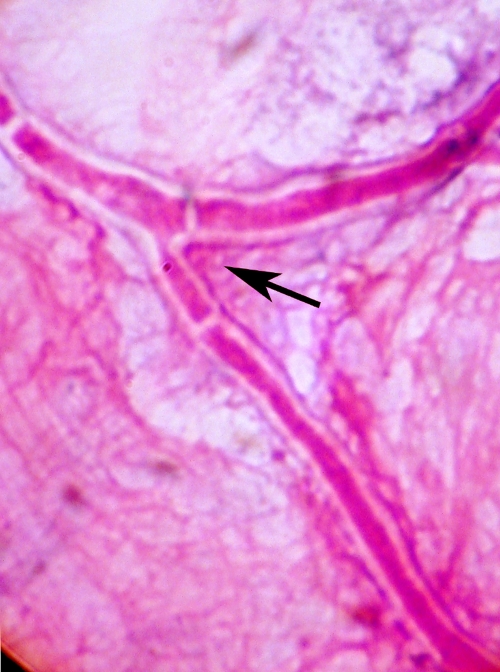
Photomicrograph showing a septate, branching hypha (arrow) of *Aspergillus flavus* in corneal scrape material from a patient with keratitis (Gram stain; 1,000×). Direct microscopic examination of corneal material by the method of Gram staining revealed the presence of fungal hyphae.

Over a 6-month period (May to October 2008), 178 patients with suppurative (suspected microbial) keratitis underwent standard clinical and microbiological investigations [29] at the Institute of Ophthalmology, Joseph Eye Hospital, Tiruchirapalli, Tamilnadu State, India. Corneal scrape material was collected from all the patients for microbiological processing; additionally, corneal biopsy material was collected from 15 patients. Written informed consent was obtained from each patient before performing corneal scraping or corneal biopsy procedures to obtain samples for microbiological investigation. The study was approved by the Institutional Review Board of Institute of Ophthalmology, Joseph Eye Hospital, Tiruchirappalli, Tamil Nadu. Filamentous fungi (55 strains) were isolated from the corneal material in 55 patients. These fungal isolates were deemed to be significant (and not mere environmental contaminants) because they were isolated on multiple culture media and because direct microscopic examination of corneal material revealed the presence of fungal hyphae (Figure 1). Ten of the 55 strains were provisionally identified as *A. flavus* based on macroscopic and microscopic characteristics (see below).

### Fungal strains

Environmental strains of filamentous fungi, provisionally identified as *A. flavus* based on macroscopic and microscopic characteristics (see below), were isolated on plates of Sabouraud glucose neopeptone agar (SDA; HiMedia,  Mumbai, India) by a settle-plate technique [30] from farming areas in and around Tiruchirappalli, India.Clinical strains of *A. flavus* were isolated as described earlier.

### Culture conditions

Soon after primary isolation, both clinical and environmental strains were subcultured once onto SDA or potato dextrose agar (PDA) slants and incubated at 25–30 °C. After growth was obtained, they were stored at 4 °C until analysis.

### Identification of fungal isolates

Isolates preliminarily identified as *A. flavus*, based on macroscopic and microscopic morphology on SDA and PDA, were subcultured to Czapek-Dox agar (CDA). The identification of *A. flavus* was made based on gross colony morphology and color and on microscopic features (magnification of 100× and 400×) in lactophenol cotton blue-stained wet mounts [1].

### Molecular identification of fungal isolates

#### DNA Extraction

DNA was extracted from 72 h-cultures of the isolates grown in Sabouraud glucose-neopeptone broth, essentially as described by Kumar et al. [31]. The cultures were centrifuged at 5000× g for 5 min, and the resulting pellets were suspended in STES buffer (SDS-1%, Tris HCl-0.2 mol/l, EDTA-0.01 mol/l, NaCl-0.5 mol/l,); glass beads were then added in a 1:1 ratio, and rigorous vortexing was done for 10 min. The cell debris was removed by centrifugation followed by extraction with phenol, chloroform and isoamyl alcohol in a ratio of 25:24:1. To the aqueous phase, 100 µg of proteinase K were added and incubated at 55 °C for 15 min, followed by extraction with a 0.5 volume of chloroform. DNA was precipitated with equal volumes of ice-cold isopropyl alcohol, and the pellets were washed with 70% ethanol and suspended in DNase/RNase-free water. The purity of the DNA was checked using both agarose gel electrophoresis and UV spectrophotometric analysis, that is, the A260/ A280 ratio.

#### DNA amplification

The ITS2 region of each test strain was amplified using the primers ITS3: 5′-GCT CGA TGA AGA ACG CAG C-3′ and ITS4: 5′-TCC TCC GCT TAT TGA TAT GC-3′ [32]. The PCR reaction was performed with a total reaction volume of 50 µl consisting of PCR buffer (1×), 0.2 mmol each of dATP, dGTP, dCTP, and dTTP, 0.5 µM of each primer and 1.5 µl of Taq DNA polymerase. After initial denaturation at 95 °C for 15 min, 30 cycles of amplification (denaturation at 95 °C for 30 s, annealing at 50 °C for 1 min and extension at 72 °C for 1 min) and a final extension at 72 °C for 2 min were performed in a thermocycler (Eppendorf, Hamburg, Germany).

#### Purification and sequence analysis

The amplicons were purified by Mini Elute gel extraction kit (Qiagen, Hilden, Germany). DNA sequencing was performed at Genei (Bangalore, India). The sequencing results were used to perform a) pairwise nucleotide sequence comparisons with 15 reference *Aspergillus* strains and b) DNA alignments by CLUSTALW. In addition, comparative GenBank sequence analysis for the identification and differentiation of the species of the study fungal strains was performed by an advanced, non-gapped BLAST search with no filtering for low complexity. The 5.8S-ITS2- 28S gene complex sequences of all the isolates were submitted to GenBank, and accession numbers were provided (Table 1).

**Table 1:** Accession numbers for the sequences of the clinical and environmental strains of *Aspergillus flavus*.

**Sample number**	**Isolate ID**	**GenBank accession number**
1	C1	HM003455
2	C2	HM003456
3	C3	HM003457
4	C4	HM003474
5	C5	HM003473
6	C6	HM003458
7	C7	HM003459
8	C8	HM003460
9	C9	HM003461
10	C10	HM003462
11	E1	HM003463
12	E2	HM003464
13	E3	HM003465
14	E4	HM003466
15	E5	HM003467
16	E6	HM003468
17	E7	HM003469
18	E8	HM003470
19	E9	HM003471
20	E10	HM003472

### Assessment of aflatoxin production in culture

The presence of aflatoxin was sought in culture filtrates and mycelial homogenates of each clinical and environmental strain of *A. flavus.* Each fungal strain was first subcultured onto slopes of SDA and incubated at 25–30 °C for 72 h for growth and sporulation. Conidia were harvested with physiologic saline containing 0.04% Tween-80 and suspensions of conidia were prepared to contain approximately 1×10^5^ CFU/ml. One ml of each conidial suspension was then inoculated into 150 ml of sterile glucose-salt media [11] and incubated at 25–30 °C for 14 days.

### Preparation of culture filtrates and mycelial homogenates

To prepare culture filtrates, each 14-day-old broth culture was successively filtered (Whatman No. 541 and Whatman No. 1 filter paper [Sigma Chemical Co., St. Louis , MO]) and then centrifuged at 17,000× g for 30 min at 4 °C in a cooling centrifuge (Heraeus, Hanau, Germany) to yield a supernatant which was then filtered through a 0.45 μm pore size membrane filter (Millipore, Bangalore, India) to remove any contaminating material, including fungal conidia and bacteria. To prepare mycelial homogenates, mycelial mats collected from the 14-day-old broth cultures were ground intermittently for 30 min with a small amount of sterile physiologic saline using sterile ground glass in a mortar and pestle under ice-cold conditions. The fully-ground material was then extracted with sterile saline and centrifuged at 17,000× g for 30 min at 4 °C to remove glass and particulate matter; the resulting supernatant was collected for analysis. The culture filtrates and mycelial homogenates thus prepared were screened for the presence of aflatoxin.

### Screening for aflatoxin production

The presence of aflatoxin in each culture filtrate and mycelial homogenate was determined by thin-layer chromatography (TLC) using a standard method [33] with some modifications. Each culture filtrate or mycelial homogenate was, in succession, extracted with acetone, filtered (Whatman No. 1 [Sigma Chemical Co.]), extracted with chloroform in a separating funnel for 3 min, filtered, passed through anhydrous sodium sulfate and concentrated at 60 °C to near dryness. The residue was re-suspended in chloroform and spotted in duplicate on 20×20 cm TLC silica gel plates (Merck, Darmstadt, Germany), which were developed in chloroform:methanol (98:2). Aflatoxin spots were visualized under ultraviolet light at 365 nm. Standard aflatoxin B1 (Sigma Chemical Co., St. Louis, MO) was used for comparison in each run. All experiments were performed at least twice.

### Quantification of aflatoxin

Aflatoxin detected by the screening process was quantified by the method of Nabney and Nesbitt [34]. The silica gel containing the aflatoxin band was scraped off from the TLC plate and extracted with cold methanol for 3 min. The methanol was then filtered off and the silica gel was washed 5 times with fresh methanol, the combined methanolic filtrate being brought up to 5 ml, and the ultraviolet absorption spectrum of the methanolic solution was then recorded. The difference between the optical density of methanolic filtrate at 363 nm and that at 420 nm was determined. This difference was then divided by the extinction coefficient (19,800) of aflatoxin B1, and the resulting figure was multiplied by the molecular weight of aflatoxin B1 (312) to obtain the concentration of aflatoxin.

### Determination of other characteristics in the fungal strains

In addition to possible aflatoxin-producing potential, each strain was tested for formation of sclerotia and production of a beige ring in culture. To assess formation of sclerotia, 2 μl of each conidial suspension (prepared as described earlier) were inoculated at a single point at the center of Petri dishes (90 mm) containing CDA and incubated at 30 °C for 7 days in the dark. To assess formation of a beige ring, the conidial suspensions were inoculated onto Petri dishes containing yeast extract sucrose agar (2% yeast extract; 20% sucrose; 2% agar) supplemented with methyl β-cyclodextrin (0.6%) and sodium desoxycholate (0.3%) (YESD medium [35],  and incubated at 30 °C for 3 days in the dark.

### Statistical analysis

The difference between the mean aflatoxin concentration in 20 culture filtrate or mycelial homogenate samples of clinical strains and that in 20 similar samples of environmental strains was analyzed by the Student “t” test. The χ^2^ test [36] was applied wherever relevant to analyze the statistical significance of differences in proportions.

## Results

Salient information regarding the 10 patients with keratitis from whom strains of *A. flavus* were isolated is provided in Table 2. The two aflatoxin-nonproducing strains were isolated from corneal material from the left eye in two males, while the eight aflatoxin-producing strains were isolated from corneal material from three males and five females (from the left eye of four patients and the right eye of four patients). Most of the corneal ulcers from which toxin-producing strains were isolated tended to be moderate in size to total ulcers. With reference to the outcome of keratitis in two patients from whom toxin-nonproducing strains were isolated, the lesion was active when last seen in one patient (this patient was lost to follow-up) while the keratitis worsened slowly over a 3-month period resulting in corneal perforation and finally healing in the other patient. In the case of eight patients from whom toxin-producing strains were isolated, there was complete healing and recovery of visual acuity in one patient and rapid worsening of the keratitis in four patients (resulting in therapeutic keratoplasty in two), with the other three patients being lost to follow-up. Composite information regarding different characteristics of the 20 (clinical and environmental) strains of *A. flavus* strains used in the study is provided in Table 3.

**Table 2:** Clinical profile of patients with culture-proven keratitis due to *Aspergillus flavus*.

** **	**Microbiology details**				**Clinical details**					
**Serial number**	**Direct Microscopy**	**Culture**	***A. flavus* Strain ID**	**Toxin Produced**	**Age & Sex**	**Affected eye**	**Ulcer Details**	**Previous treatment**	**Treatment**	**Outcome**
1	LPCB 3+ Gram3+	*Aspergillus flavus* & *Enterobacter* sp.	C1	Yes	32/F	RE	Medium –sized ulcer with hypopyon	Antibio Flucon TEM	Nata Moxi Oral KC	Active even after 10 days treatment
2.	LPCB 4+ Gram 2+	*Aspergillus flavus* only	C2	No	39/M	LE	Medium –sized ulcer with hypopyon	Antibio Antivirals	Nata 18 days	Active even after 10 days treatment
3.	LPCB:occ. Gram:occ.	*Aspergillus flavus* & *S. aureus*	C3	Yes	39/M	RE	Small ulcer	Antibiotics	NA	NA
4.	LPCB 3+ Gram 2+	*Aspergillus flavus* only	C4	Yes	55/M	LE	Medium –sized ulcer	NA	NA	NA
5.	LPCB 3+ Gram 2+	*Aspergillus flavus* only	C5	Yes	55/F	RE	Medium –sized ulcer	Antibiotics	NA	NA
6.	LPCB occ. Gram occ.	*Aspergillus flavus* only	C6	Yes	53/M	RE	Small ulcer	Antifungal	Nata	Healed in 20 d
7.	LPCB 4+ Gram 3+	*Aspergillus flavus* only	C7	No	60/M	LE	Small ulcer	Antibiotics Antifungal	Nata & KC	Slow progression, perforated & then healed
8.	LPCB 3+ Gram 3+	*Aspergillus flavus* only	C8	Yes	70/F	LE	Total ulcer	Antibiotics	Nata	NA
9#	LPCB +* Gram +	*Aspergillus flavus* & *Enterobacter* sp.	C9	Yes	50/F	LE	Total Ulcer	Antibiotics Antifungal	Nata	TPK
10.	LPCB 2+* Gram 2+	*Aspergillus flavus* only	C10	Yes	50/F	LE	Total ulcer	N.A.	NA	TPK

The sharp (hash mark) indicates that initially, a corneal scraping was obtained from this patient for microbiological workup which grew *Aspergillus flavus*; aflatoxin-producing activity was not assessed for this strain. The asterisk indicates corneal button sample LPCB- Lactophenol Cotton Blue Mount, Gram- Gram staining of the corneal material by direct microscopy; 4+ represents the presence of abundant fungal hyphae, 3+ represent the presence of more number of fungal hyphae, 2+ represents the presence of moderate number of fungal hyphae, + represents the presence of less number of fungal hyphae, occ represents the presence of occasional fungal hyphae in the specimen from the patients with suppurative keratitis; F- female, M- Male; Aff.Eye- affected eye; RE- right eye, LE- Left Eye; Prev. ttmt- previous treatment; NA: Data not available; Ttmt- treatment: Nata- natamycin, Moxi- moxifloxacin, KC-ketoconazole ; TPK- therapeutic penetrating keratoplasty.

**Table 3:** Characteristics of *Aspergillus flavus* strains isolated from clinical and environmental sources.

**Serial number**	**Isolate ID**	Aflatoxin detected *(positives/number of samples)	Aflatoxin concentration (ppb)**		**Sclerotia formed#**	**Beige ring formed##**
			**Culture filtrate**	**Mycelial homogenate**		
1	C1	Yes (2/2)	315.0	385.0	Yes	Yes
2	C2	No (0/2)	0	0	No	No
3	C3	Yes (2/2)	203.5	232.5	Yes	Yes
4	C4	Yes (2/2)	545.0	661.8	Yes	Yes
5	C5	Yes (2/2)	321.3	362.4	Yes	Yes
6	C6	Yes (2/2)	475.1	504.2	Yes	Yes
7	C7	No (0/2)	0	0	No	No
8	C8	Yes (2/2)	241.2	252.1	Yes	No
9	C9	Yes (2/2)	302.7	362.4	No	Yes
10	C10	Yes (2/2)	311.4	391.2	Yes	Yes
Total	10 clinical Isolates	Yes (16/20) No (4/20)	Mean aflatoxin concentration 366.7±125.4 ppb		Yes=7 No=3	Yes=7 No=3
11	E1	Yes (2/2)	312.5	420.5	Yes	Yes
12	E2	No (0/2)	0	0	No	No
13	E3	No (0/2)	0	0	No	No
14	E4	Yes (2/2)	210.5	211.2	Yes	Yes
15	E5	No (0/2)	0	0	No	Yes
16	E6	Yes (2/2)	310.5	330.9	No	Yes
17	E7	No (0/2)	0	0	No	No
18	E8	Yes (2/2)	311.33	345.0	No	Yes
19	E9	No (0/2)	0	0	No	No
20	E10	No (0/2)	0	0	No	No
Total	10 environmental isolates	Yes (8/20) No (12/20)	Mean aflatoxin concentration 306.5±125.4 ppb		Yes=2 No=8	Yes=5 No=5

C1 to C10=Clinical isolates of Aspergillus flavus (from patients with keratitis); E1 to E10=Environmental isolates of Aspergillus flavus (from farming areas). The asterisk indicates detected by screening culture filtrates & mycelial homogenates on thin layer chromatography plates. the double asterisk indicates aflatoxin concentration in parts per billion (ppb). The sharp (hash mark) indicates sclerotia size(diameter) >400 µm. The double sharp indicates detected by direct visual examination Statistical analysis (χ^2^ test [Pearson's]; degree of freedom=1). a) Aflatoxin detected in 16 /20 samples from clinical strains versus 8/20 samples from environmental strains; χ^2^=6.667; p=0.0098. b) % of clinical versus % of environmental strains forming sclerotia in culture: χ^2^=5.051 (p=0.0246). c) % of aflatoxin-producing versus % of aflatoxin-nonproducing strains forming sclerotia in culture: χ^2^=10.909 (p=0.0009). d) % of clinical versus % of environmental strains forming a beige ring in culture χ^2^=0.833 (p=0.3613). e) % of aflatoxin-producing versus % of aflatoxin- nonproducing strains forming a beige ring in culture: χ^2^=12.535 (p=0.0003).

**Figure 2:**
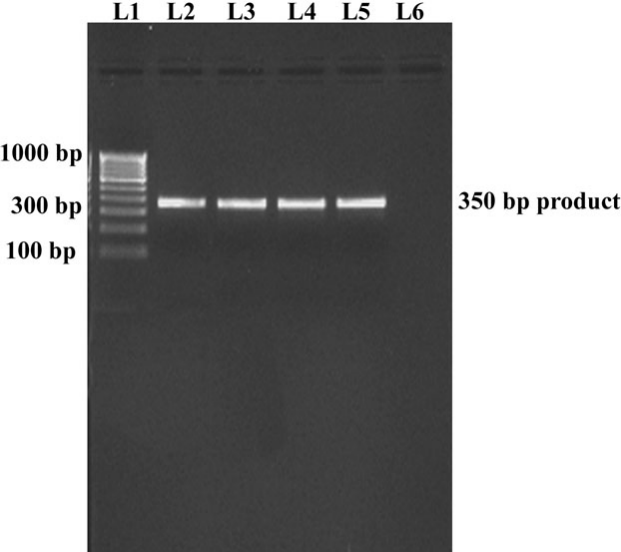
Gel picture showing the PCR amplified product (350 bp) of the three isolates of *Aspergillus flavus.* Internal transcribed spacer 2 was used as a target region for DNA amplification. The lane L1 was loaded with 100 bp DNA Ladder; the lanes L2, L3, L4, L5 were loaded with 10 μl of the amplified product from the test strains C1, C2, E1, E2, respectively, in 1.5% agarose gels. Lane L6 was loaded with a negative control to rule out false-positive results.

During gel electrophoresis of the amplified products, no significant differences in the length of the amplicons (350 bp) were observed among the fungal strains (Figure 2). The ITS2 region sequences of the study strains were compared by BLAST analysis with those of selected reference strains of *A. flavus* (ATCC 11497, NRRL 4998, NRRL 4822; NRRL 458, NRRL 3751, NRRL 3518, and NRRL2097); all the study strains exhibited 100% identity with the molecular siblings. Less than 91% similarity was seen when comparing the sequences of the study strains to those of other *Aspergillus* species, except that of *Aspergillus oryzae*, which revealed a sequence similarity of 99%. DNA sequence alignments of the study strains against the sequences of other selected non-*Aspergillus* pathogenic fungi in the GenBank database revealed marked variability. Finally, the sequences of the study strains were subjected to Blastn in GenBank; the results obtained were similar to those obtained in the preceding steps. The assigned sequence accession numbers are listed in Table 1.

**Figure 3:**
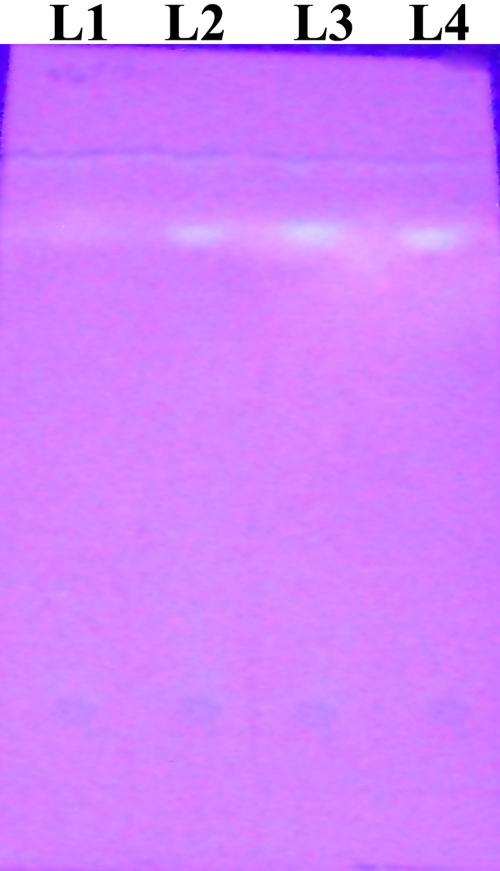
Thin layer chromatograms of culture filtrates of two clinical and one environmental isolate of *Aspergillus flavus.* L1: aflatoxin B1 was not detected in the extract from the culture filatrate of test strain C2 (aflatoxin-non producing clinical strain). L2: aflatoxin B1 was detected in the extract from the culture filtrate of test strain C1 (aflatoxin-producing clinical strain). L3: aflatoxin B1 was detected in the extract from the culture filtrate of the test strain E1 (aflatoxin-producing environmental strain). L4: aflatoxin B1 standard was loaded for confirmation.

Aflatoxin (only B1) was detected by TLC (Figure 3) in 16 (80%) of 20 samples (culture filtrate or mycelial homogenate) of the clinical strains and in eight (40%) of 20 samples of the environmental strains (Table 2; χ2=6.667; p=0.0098). The mean quantum of aflatoxin B1 detected in the samples of the clinical strains (366.7±125.4 ppb) was higher than that detected in the samples of the environmental strains (306.5±69.1 ppb), but this difference was not statistically significant.

**Figure 4:**
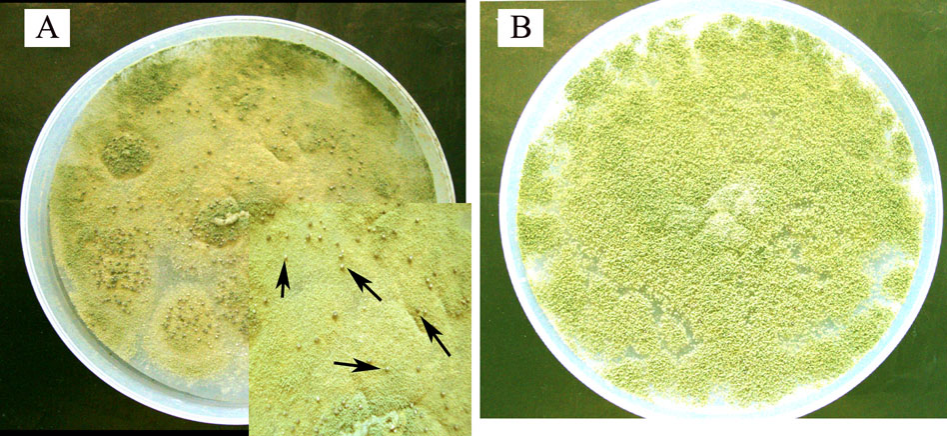
Seven day-old growth of a clinical isolate of *Aspergillus flavus* on Czapek-Dox agar incubated at 30 °C. **A**: Formation of sclerotia is noted in the culture (arrows in inset). Formation of sclerotia was noted significantly more frequently in aflatoxin-producing strains than in aflatoxin-nonproducing strains. **B**: Seven day-old growth of another clinical isolate of *Aspergillus flavus* on Czapek-Dox agar incubated 30 °C. This isolate has not formed sclerotia in culture.

A significantly higher (χ2=5.051; p=0.0246) proportion of clinical (70%) than environmental (20%) isolates produced sclerotia in culture (Figure 4A,B), the size of the sclerotia exceeding 400 μm in all instances (indicating that the isolates producing sclerotia were all `L’ types). Nine (75%) of the 12 aflatoxin-producing clinical or environmental isolates formed sclerotia in culture compared to none of the eight aflatoxin-nonproducing strains (χ2=10.909; p=0.0009).

**Figure 5:**
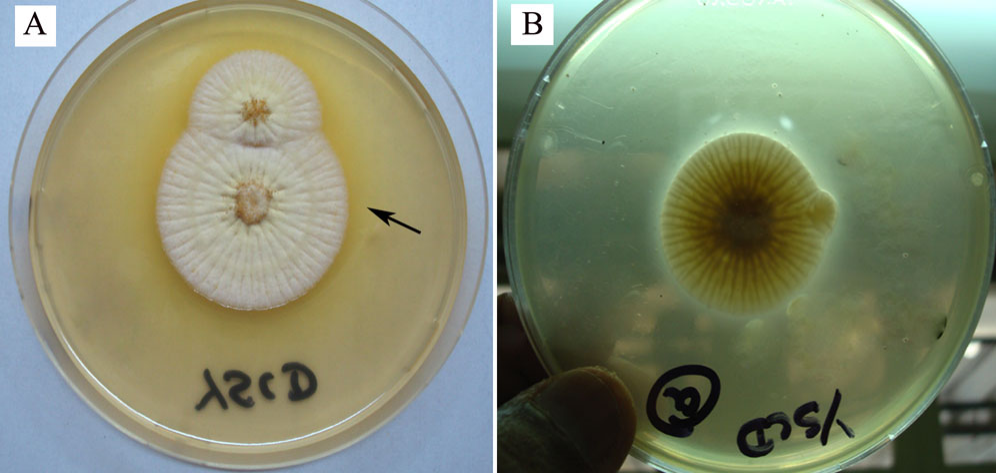
Three day-old growth of the isolate of *Aspergillus flavus* on yeast extract sucrose agar (supplemented with methyl β-cyclodextrin and sodium desoxycholate), incubated at 30 °C. **A**: Three day-old growth of a clinical isolate of *Aspergillus flavus* on yeast extract sucrose agar (supplemented with methyl β-cyclodextrin and sodium desoxycholate), incubated at 30 °C. A beige ring (arrow) has formed around the colony. Formation of a beige ring was noted significantly more frequently in aflatoxin-producing strains than in aflatoxin-nonproducing strains. **B**: Three day-old growth of an environmental isolate of *Aspergillus flavus* on yeast extract sucrose agar supplemented with methyl β-cyclodextrin and sodium desoxycholate), incubated at 30 °C. There is no beige ring around the colony growth.

Seventy percent of the clinical isolates and 50% of the environmental isolates produced a beige ring in culture (Figure 5A,B). Eleven (92%) of 12 aflatoxin-producing clinical or environmental strains and one (12.5%) of eight aflatoxin-non producing strains formed a beige ring in culture (χ2=13.0; p<0.001).

## Discussion

Weakening of specific immunological and non-specific host defenses may predispose to *Aspergillus* infections in debilitated and immunocompromised patients in hospitals. Thermotolerance, the ability to grow in anaerobic environments and the ability to produce certain proteolytic enzymes, such as elastases, are putative virulence factors that allow fungi to elude specific host defenses [37-41]. However, other factors need to be elucidated.

Aflatoxins are secondary metabolites produced by members of the *Aspergillus flavus* complex, principally *A. flavus* and *A. parasiticus* [1], and cause illness and disease in poultry and domestic animals [41]. However, little is known about production of aflatoxins by clinical isolates of *A. flavus* (strains isolated from lesions in humans). This is an aspect that requires investigation because of possible therapeutic implications. If an *A. flavus* strain isolated from a human lesion is found to possess aflatoxin-producing ability, it may be necessary to treat the lesion with not only standard antifungal therapy, which act only on the fungus, but also with molecules that suppress the production, or neutralize the deleterious effects, of aflatoxins.

We identified the clinical and environmental fungal strains used in the present study by both morphological and molecular methods, since molecular approaches provide more rapid and objective identification than do traditional phenotypic methods. Constituents of the ITS region have been used as targets to identify species, because they generally display sequence variation between species, but only minor, or no, variation within strains of the same species [32,42-45]. We targeted the ITS2 region for amplification and direct sequencing. Direct sequencing, followed by comparative GenBank analysis, is considered to be one of the most reliable methods for the identification of species [32,43]. We used comparative GenBank analysis to confirm that the clinical and environmental strains were, in fact, *A. flavus* strains. Several targets for the molecular identification of aspergilli have been investigated in other studies including the mitochondrial cytochrome b gene [46], a putative aflatoxin pathway regulatory gene (*aflR*), the DNA topoisomerase gene [47], the β-tubulin gene [45] and various rRNA gene regions [32]. But the most reliable target investigated is ITS region [32], and hence we selected the ITS2 region as the target for the molecular identification of the fungal isolates.

*A. flavus* is the second most frequent cause of invasive aspergillosis [1,4] and has also emerged as a predominant pathogen in patients with fungal sinusitis and fungal keratitis in several institutions worldwide [6-8,48]. Conidia in the environment serve as the major source of inoculum for *Aspergillus* species (including *A. flavus*) that cause opportunistic infections in plants and animals, including humans [49]. Kosalec and Pepeljnjak [50] detected aflatoxin B1 in seven (23%) and aflatoxin G1 in one (3%) of 30 clinical isolates of *A. flavus* (from immunocompromised patients in a hematological unit), and also detected aflatoxins B1 and G1 in 11 (37%) and one (3%) of 30 environmental isolates of *A. flavus*. Considering this, in the present study, the isolates from the cornea and the environment should have exhibited roughly equal frequencies of aflatoxin production; instead, we observed that aflatoxin B1 was detected in 80% of culture filtrate or mycelial homogenate samples of clinical *A. flavus* strains, but in only 40% of such samples of environmental *A. flavus* strains (Table 1). Production of aflatoxin in vitro by strains of *A. flavus* isolated from patients with keratitis has not previously been reported.

The external environment is the natural habitat of *A. flavus;* therefore, *A. flavus* strains causing keratitis by infecting the cornea would be in an abnormal setting. In such a situation, the observed high frequency of aflatoxin production by the clinical *A. flavus* strains in the present study possibly represented a response to pressures (antifungal chemotherapy, toxic factors released from corneal epithelial cells, or infiltrating inflammatory cells) that are not conducive to an ideal existence. A completely contradictory explanation is that the corneal tissue, taken as a whole, is actually conducive to aflatoxin biosynthesis by infecting *A. flavus* strains; for example, the normal corneal temperature of 33 °C to 34 °C [51,52] may stimulate synthesis of aflatoxin. In this context, it is interesting to note that in two different studies [13,53], the optimal temperature for aflatoxin production by *A. flavus* was found to be 30 °C, with no toxin production at 10 °C [54] or at 20 °C and 37 °C [13,53]; in addition, a complex interaction of temperature, water activity, incubation period, and substrate has been found to influence the relative concentrations of aflatoxins produced by *A. flavus* [13].

Thus, in the present study, the infected corneal tissue possibly provided either an unfavorable setting or a favorable setting for growth of *A. flavus*, the end-result in either case being activation of regulatory fungal genes such as *aflR* and *aflJ* which are now considered to be involved in the regulation of aflatoxin production [55-57]; this effect possibly persisted even after the fungus was isolated in culture from corneal scrape material, and subcultured one more time to culture media. Interestingly, structural genes, such as *pksA* and *norA*, are also considered to play an important role in aflatoxin production; deletion of these genes may lead to complete cessation of aflatoxin production [55].These hypotheses need to be confirmed by a) detection of aflatoxin in corneal tissue infected by aflatoxin-producing strains of *A. flavus* and b) demonstrating an activation of the fungal genes governing aflatoxin biosynthesis upon transferring aflatoxin-non producing strains of *A. flavus* from the natural environment to corneal tissue. These aspects will form the basis of future studies.

When we analyzed the salient information regarding the 10 patients with keratitis from whom strains of *A. flavus* were isolated, we were unable to discern definite patterns to correlate aflatoxin production by the *A. flavus* strain and outcome of therapy. Thus, a larger series of patients probably needs to be examined. However, it was interesting to note that the corneal ulceration tended to be moderate in size to total corneal ulcers in six of the eight patients from whom toxin-producing strains were isolated. Toxin production by the fungal strain may have contributed to the severity of the lesions observed in these six patients, and the consequent poor response to medical therapy. Mori et al. [58] reported the production of aflatoxins in vivo and in vitro by the isolates of *A. flavus* from the post mortem lung sample from a patient with systemic aspergillosis and suggested a possible role of aflatoxins in damaging the immune system through their toxic effects.

Most asexual *Aspergillus* species, including *A. flavus,* form resistant structures called sclerotia [50] which survive environmental extremes even over long periods and form fresh mycelia when conditions are favorable, thus re-establishing the infection process. There appears to be a relationship between formation of sclerotia and aflatoxin production in culture. Strains of *A. flavus* that form many small sclerotia in culture (`S’ types) produce both B- and G-type aflatoxins [59,60], whereas those that form fewer, large sclerotia in culture (`L’ types) produce only aflatoxin B1 [61]. Bennet et al. [62] found no correlation between aflatoxin production and sclerotial production among 14 isolates of *A. flavus* and *A. parasiticus* whereas Cotty [63] reported a positive correlation between high aflatoxin production and presence of small (<400 μm) sclerotia. In the present study, nine (75%) of the 12 aflatoxin-producing (clinical or environmental) strains of *A. flavus* and none of the eight aflatoxin nonproducing strains formed sclerotia in culture (p=0.0009). Our observations thus suggest that there is a definite relationship between formation of sclerotia and production of aflatoxin by *A. flavus*, and that sclerotia formation may be a reliable marker of aflatoxin production by an *A. flavus* strain.

Aflatoxin-producing strains of *A. flavus* have also been reported to form a yellow pigment in culture [64-66]. In the present study, 92% of the aflatoxin-producing (clinical and environmental) strains and only 12.5% of the aflatoxin-nonproducing strains of *A. flavus* were found to form a beige ring in culture (p=0.0003). These results suggest that the presence of a beige ring (visible to the naked eye under natural light) surrounding colonies of an *A. flavus* strain in YESD medium may serve as a marker of aflatoxin-producing potential.

In conclusion, the results of the present study suggest that aflatoxin production occurs significantly more frequently in strains of *A. flavus* isolated from patients with keratitis than it does in strains of *A. flavus* isolated from the environment. The reasons for this phenomenon are unclear and require further investigation; possibly, the corneal tissue provides either an unfavorable or a favorable setting for growth of *A. flavus*, the end- result in either case being a stimulus for aflatoxin production. This observation has therapeutic implications, since it may be necessary to treat patients having keratitis due to *A. flavus* with molecules that suppress production, or neutralize the deleterious effects, of aflatoxins. Formation of sclerotia in CDA and of a beige ring in YESD medium by a strain of *A. flavus* may serve as markers of its aflatoxin-forming potential. The potential to produce aflatoxin may contribute to the severity of corneal lesions caused by strains of *Aspergillus flavus*.
